# Long term clinical outcomes of patients with ischemic stroke in primary care – a 9-year retrospective study

**DOI:** 10.1186/s12875-021-01513-w

**Published:** 2021-08-07

**Authors:** Jinghao Han, Yue Kwan Choi, Wing Kit Leung, Ming Tung Hui, Maria Kwan Wa Leung

**Affiliations:** Department of Family Medicine, New Territories East Cluster, Hospital Authority, Hong Kong, China

**Keywords:** Stroke, Cardiovascular disease (CVD) risk, Secondary prevention, Statin, Primary care

## Abstract

**Background:**

We aim to document the long-term outcomes of ischemic stroke patients and explore the potential risk factors for recurrent cardiovascular events and all-cause mortality in primary care.

**Methods:**

A retrospective cohort study performed at two general out-patient clinics (GOPCs) under Hospital Authority (HA) in Hong Kong (HK). Ischemic stroke patients with at least two consecutive follow-up visits during the recruitment period (1/1–30/6/2010) were included. Patients were followed up regularly till the date of recurrent stroke, cardiovascular event, death or 31/12/2018. The primary outcome was the occurrence of recurrent cerebrovascular event including transient ischemic stroke (TIA), ischemic stroke or hemorrhagic stroke. The secondary outcomes were all-cause mortality and coronary artery disease (CAD). We fit cox proportional hazard model adjusting death as competing risk factor to estimate the cause-specific hazard ratio (csHR).

**Results:**

A total of 466 patients (mean age, 71.5 years) were included. During a median follow-up period of 8.7 years, 158 patients (33.9%) died. Eighty patients (17.2%) had recurrent stroke and 57 (12.2%) patients developed CAD. Age was an independent risk factor for recurrent stroke, CAD and death. Statin therapy at baseline had a protective effect for recurrent stroke (csHR = 0.476; 95% confidence interval [CI] 0.285–0.796, P = 0.005) after adjusting death as a competing risk factor and all-cause mortality (HR = 0.693, 95% CI 0.486–0.968, P = 0.043). In addition, female sex, antiplatelet and a higher diastolic blood pressure (DBP) at baseline were also independent predictors for survival.

**Conclusions:**

Long term prognosis of ischemic stroke patients in primary care is favorable. Use of statin was associated with a significant decrease in stroke recurrence and mortality. Patients who died had a significant lower DBP at baseline, highlighted the need to consider both systolic and diastolic blood pressure in our daily practice.

## Background

Stroke is the second commonest cause of death worldwide [1] and the 4^th^ leading cause of death in Hong Kong [2]. As the aging population is rapidly increasing in HK, stroke will remain as a huge burden to patient’s families and the entire health care system. Over 80% of all strokes cases are ischemic stroke. Ischemic stroke patients are at high risk for stroke recurrence and other cardiovascular events [3–6]. The risk of recurrent stroke is about 12–13% in the first year [3–5], 18.3–35.5% at 5 years [3–6] and 40–51% at 10 years [4–7].

Effective measures for secondary prevention of stroke includes lifestyle modifications, vascular risk factor control and use of antiplatelet and anticoagulant drugs. The clinical protocol of secondary stroke prevention for ischemic stroke patients [Department of Family Medicine, New Territories East Cluster (NTEC), HA] was summarized as follows [8–11]:

1. Record smoking status, amount of exercise and body mass index (BMI), offer advice
2. Check blood pressure (BP) at each visit
3. BP < 140/90 mmHg for patients without diabetes
4. BP < 130/80 mmHg for patients with diabetes
5. Check fasting blood glucose and lipids annually
6. Aim for hemoglobin A1c (HbA1_C_) < 7% for patients with diabetes mellitus (DM)
7. Hyperlipidemia: aim for low-density lipoprotein (LDL) < 1.8 mmol/L
8. Antiplatelet agents for patients with non-cardioembolic ischemic stroke unless contraindicated, options including aspirin /aspirin plus extended- release dipydridamole/ Clopidogrel
9. Warfarin or novel oral anticoagulant for patients with atrial fibrillation (AF) unless contraindicated.

Family physicians play a fundamental role in secondary stroke prevention management because stroke patients need lifelong control of vascular risk factors. Our previous study found the implementation of secondary stroke prevention program in primary care could improve control of cardiovascular risk factors including BP, HbA1c and LDL levels [11]. However, whether it has an impact on the long term clinical outcomes of ischemic stroke patients remains unknown. In addition, local data on survival and recurrence of cerebrovascular events among patients with ischemic stroke in primary care is lacking.

The aim of this study was to document the long-term outcomes of ischemic stroke patients and explore the potential risk factors for recurrent cardiovascular events and all-cause mortality in primary care. The primary outcome was the occurrence of recurrent cerebrovascular events including TIA, ischemic stroke or hemorrhagic stroke. The secondary outcomes were all-cause mortality and development of CAD, respectively.

## Methods

### Study design

This was a retrospective cohort study performed at two major GOPCs (Lek Yuen & Ma On Shan family medicine center, NTEC, HA), which served a population of 630,000 in Shatin District (8.9% of HK population).

#### Inclusion and exclusion criteria

Subjects were identified from the Clinical Management System (CMS) database of the HA. Inclusion criteria were (1) Age ≥ 18; (2) Non-acute ischemic stroke patients with coding by International Classification of Primary Care, 2nd edition (ICPC-2) K90 (stroke/cerebrovascular accident) or K91 (cerebrovascular disease); (3) Only those patients diagnosed with ischemic stroke and who had at least 2 consecutive follow-up visits in the same clinic within the recruitment period were included. Patients were excluded if 1) they had a previous history of acute coronary syndrome or revascularization procedure of coronary artery; 2) hemorrhagic stroke; 3) acute ischemic stroke within 4 weeks of onset; 4) being followed up by medical specialists only. The recruitment period lasted for 6 months from 1/1/2010 -30/6/2010. Ethical approval of this study was granted by The Joint Chinese University of Hong Kong – New Territories East Cluster Clinical Research Ethics Committee (Reference No. 2019.252).

#### Outcome measures

The primary outcome was the recurrence of fatal and non-fatal cerebrovascular events including TIA, ischemic stroke or hemorrhagic stroke. The secondary outcomes were all-cause mortality and CAD, respectively. If death or a recurrent cerebrovascular or cardiac event was recorded as occurring simultaneously, one event was included into the analysis. The incidence of cardiovascular events was identified by the International Classification of Diseases, Ninth Edition, Clinical Modification (ICD-9-CM) and ICPC-2 codes from the CMS. The earliest date of diagnosis with ICD-9-CM of 430.x to 438.x was the time of recurrent stroke event. The earliest date of diagnosis with ICPC-2 of K74 to K76 or ICD-9-CM of 410.x, 411.x to 414.x, 36.07, 36.10 to 36.14 was defined as the time of development of CAD (angina pectoris, myocardial infarction (MI), percutaneous coronary intervention or coronary artery bypass graft surgery). The recurrent cardiovascular events and death causes were further identified by reviewing of the medical discharge summary records. Indefinite information about the cause of death was labelled as unknown.

#### Baseline covariates

Baseline information of patients including socio-demographics, clinical parameters, and regular medications were retrieved from CMS. The socio-demographics of patients included gender, age and smoking status. The patient was considered a smoker if he/she currently smoked. Clinical parameters including BMI, BP, lipid profile and HbA1c if diabetic were extracted from CMS. Co-morbidity including obesity, HT, DM were retrieved by ICPC-2 code from the CMS. Hypertension (HT) was defined as a history of systolic blood pressure (SBP) ≥ 140 mmHg or diastolic blood pressure (DBP) ≥ 90 mmHg (SBP ≥ 130 mmHg or DBP ≥ 80 mmHg among diabetic patients), or a history of treatment with antihypertensive medication. The diagnosis of DM was established with any of the following criteria: 1) when a patient presented with classic symptoms of hyperglycemia and has a random plasma glucose value of ≥ 11.1 mmol/L; 2) In an asymptomatic individual with fasting blood glucose level ≥ 7.0 mmol/L, HbA1c ≥ 6.5%, a 2 h post-oral glucose tolerance test values of ≥ 11.1 mmol/L. In the absence of unequivocal symptomatic hyperglycemia, the diagnosis of DM could be established on a subsequent day by repeating the same test for confirmation. Obesity was defined as BMI ≥ 25.0 kg/m^2^. Treatment modalities including the use of antihypertensive agents, statins, antiplatelet and antidiabetic medications were retrieved from CMS. Statin users were defined as patients receiving statin at baseline. Non-statin user was defined as follows: 1) patient not using statin at baseline; 2) patient who stopped statin within 6 months after initiation of treatment at baseline.

#### Follow-up

The entry date was the first attendance record in GOPC clinics for stroke follow-up during the recruitment period. All patients were followed up regularly at an interval of 3 to 4 months till the date of incidence of the first recurrent stroke, cardiac event, death, the censored date of lost to follow-up or 31/12/2018. Cases were censored for recurrence if they died before experiencing a recurrent event.

#### Statistical Analysis

Data analyses were performed using the SPSS software (version 26.0 for windows). All tests were two-sided, and P values < 0.05 were regarded as statistically significant. Baseline comparisons were made with the Student’s t-test or the chi-squared test as appropriate. To test linearity of the continuous variable in a Cox model, we fit a Cox model with a linear effect and with a nonlinear effect using natural cubic splines on the log hazard scale. ANOVA likelihood ratio test was used for linearity. We fit a cox proportional hazard model adjusting death as competing risk factor for estimate the cause-specific hazard ratio. [12] Hazard ratio (HR) and its 95% confidence interval (CI) were reported for each variable within the regression model after adjusting for other confounding factors. Cumulative incidence analysis and Kaplan Meier survive curves were conducted using R package ‘cmprsk’. [13]

#### Sample size calculations

Our pilot study including 100 subjects found the recurrent stroke rate and mortality rate was 0.2 and 0.3, respectively. Given a confidence level of 95% and a relative precision of 0.2, the sample size needed for observing the expected proportion of recurrent stroke and mortality among our GOPC patients was 385 and 225, respectively. Below is the formula of sample size calculation: $\mathrm{n}={\mathrm{z}}_{1-\mathrm{\alpha }/\upvarepsilon }^{2}\frac{1-\mathrm{P}}{{\upvarepsilon }^{2}\mathrm{P}}$, where p = 0.2 (stroke recurrent rate) or 0.3 (all-cause mortality), z = 1.96, ε = 20% or 0.2.

## Results

**Fig. 1:**
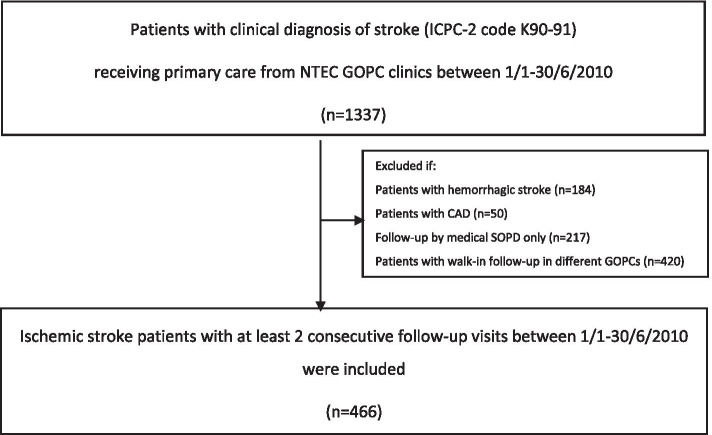
Flow Charts of Subjects

This retrospective study, which extended from 1 January 2010 to 31 December 2018, included a total of 466 patients with history of ischemic stroke under primary care (Fig. 1). The baseline characteristics of patients are shown in Table 1. The sex ratio was close to 1:1 and mean age of the cohort was 71.5 years. Mean LDL level was 2.9 mmol/L and only 187 (40.1%) patients received statin at baseline. Three patients lost follow-up because they resided outside HK. The last date of follow-up was used as censored date. Table 2 shows the observed number of recurrent stroke, cardiovascular events and all-cause death during follow-up. Eighty patients experienced recurrent stroke with 78.8% had ischemic stroke. A total of 158 patients died, among them there were 46 (29.1%) vascular death. The annual incidence rate of recurrent stroke and all-cause mortality were 1.9% and 3.8%, respectively.

**Table 1:** Baseline Characteristics of Ischemic Stroke Patients at 2010 (*n* = 466)

	Subject (*n* = 466)
Sex	
Female (%)	236 (50.6)
Male (%)	230 (49.4)
Age (Years, mean ± SD)	71.5 ± 11.2
BMI (Kg/m^2^)	24.3 ± 3.6
Current smoker (%)	37 (7.9)
HT (%)	402 (86.3)
SBP (mmHg, mean ± SD)	130.8 ± 14.9
DBP (mmHg,, mean ± SD)	70.1 ± 10.1
DM (%)	136 (29.2)
HbA1c	6.7 ± 0.9
LDL (mmol/L, mean ± SD)	2.9 ± 0.7
Statin user (%)	187 (40.1)
Atrial fibrillation (%)	5 (1.1)
Antiplatelet treatment (%)	451(96.8)

*BMI* indicates body mass index; *HT* hypertension; *SBP* systolic blood pressure; *DBP* diastolic blood pressure; *DM* diabetes mellitus; *HbA1c* hemoglobin A1c and LDL, low- density lipoprotein- cholesterol

**Table 2:** Clinical Outcomes of Ischemic Stroke Patients (*n* = 466)

	No. of Patients	
**Cardiovascular event**		
*** Recurrent cerebrovascular events*** (%)	80 (17.2)	
TIA (%)	3 (0.6)	
Ischemic stroke (%)	60 (12.9)	
Hemorrhagic stroke (%)	17 (3.6)	
*** CAD*** (%)	57 (12.2)	
**All-cause mortality** (%)	158 (33.9)	
*** Vascular death* (%)**	46 (9.9)	
Fatal recurrent ischemic stroke (%)	14 (3.0)	
Fatal recurrent hemorrhagic stroke (%)	6 (12.9)	
Fatal CAD (%)	26(5.6)	
*** Non-vascular death*** (%)	110(23.6)	
Pneumonia (%)	53 (11.4)	
Cancer (%)	48 (10.3)	
Sepsis (%)	3 (0.6)	
Renal failure (%)	2 (0.4)	
Choking (%)	4 (0.9)	
*** Unknown*** (%)	2 (0.4)	

Younger patients and statin users at baseline had a lower rate of recurrent stroke and death (Table 3). Patients who died had a significant lower DBP at baseline ( 67.6 ± 9.3 vs 71.4 ± 10.2; *P* < 0.001, Table 3). Patients who developed CAD were older (76. 6 ± 8.9 vs 70.6 ± 12.0; *P* < 0.001) and had a significantly higher SBP (134.7 ± 16.78 vs 130.2 ± 14.5; *P* = 0.03) at baseline.

**Table 3:** Baseline Characteristics of Ischemic Stroke Patients in 2010

	**Recurrent cerebrovascular event** **(*n* = 80)**	**Non-recurrent cerebrovascular event** **(*n* = 386)**	***P* value**	**CAD** **(*n* = 57)**	**Non-CAD** **(*n* = 409)**	***P* value**	**Death** **(*n* = 158)**	**Survival** **(*n* = 308)**	***P* value**
Sex									
Female (%)	38 (47.5)	198 (51.3)	0.54	32(56.1)	204 (49.9)	0.38	74 (46.8)	162 (52.6)	0.24
Male (%)	42 (52.5)	188 (48.7)		25 (43.9)	205(50.1)		84 (53.2)	146(47.4)	
Age (Years)	74.0 ± 8.8	71.0 ± 11.6	***0.03***	76.6 ± 8.9	70.6 ± 12.0	** < *0.001***	77.6 ± 8.1	68.4 ± 11.4	< 0.001*
BMI (Kg/m^2^)	24.2 ± 3.1	24.3 ± 3.7	0.91	23.6 ± 3.9	24.3 ± 3.6	0.32	23.6 ± 3.1	24.5 ± 3.8	0.06
Current smoker (%)	6 (7.5)	31 (8.0)	0.87	3 (5.3)	34 (8.3)	0.61	13 (8.2)	24 (7.8)	0.86
HT (%)	71 (88.8)	331 (85.8)	0.48	53 (93.0)	349 (85.3)	0.12	138 (87.3)	264 (85.7)	0.63
SBP (mmHg)	130.6 ± 14.9	130.8 ± 14.9	0.94	134.7 ± 16.7	130.2 ± 14.5	***0.03***	132.0 ± 16.8	130.1 ± 13.8	0.22
DBP (mmHg)	69.1 ± 9.3	70.3 ± 10.2	0.36	70.1 ± 9.9	70.1 ± 10.1	0.99	67.6 ± 9.3	71.4 ± 10.2	** < *0.001***
DM (%)	28 (35.0)	108 (28.0)	0.21	16 (28.1)	120 (29.3)	0.84	48 (30.4)	88 (28.6)	0.68
HbA1c (%)	6.7 ± 0.6	6.7 ± 1.0	0.75	6.7 ± 0.9	6.6 ± 0.8	0.32	6.7 ± 1.1	6.7 ± 0.9	0.74
LDL (mmol/L)	2.9 ± 0.8	2.9 ± 0.8	0.90	2.8 ± 0.6	2.9 ± 0.7	0.42	2.8 ± 0.8	2.9 ± 0.7	0.36
Statin user (%)	19 (23.8)	168 (43.5)	***0.001***	18 (31.6)	169 (41.3)	0.16	45 (28.5)	142 (46.1)	** < *0.001***
Atrial fibrillation (%)	0	5 (1.3)	0.59	1 (1.8)	4 (1.0)	0.48	1 (0.6)	4 (1.3)	0.67
Antiplatelet treatment (%)	78 (97.5)	373 (96.6)	0.99	56 (98.2)	395(96.6)	0.50	150 (94.9)	301(97.7)	0.11

*CAD* indicates coronary artery disease; *BMI* body mass index; *HT* hypertension; *SBP* systolic blood pressure; *DBP* diastolic blood pressure; *DM* diabetes mellitus; *HbA1c* hemoglobin A1c and *LDL* low- density lipoprotein- cholesterol

^*^Equal variances not assumed

**Fig. 2:**
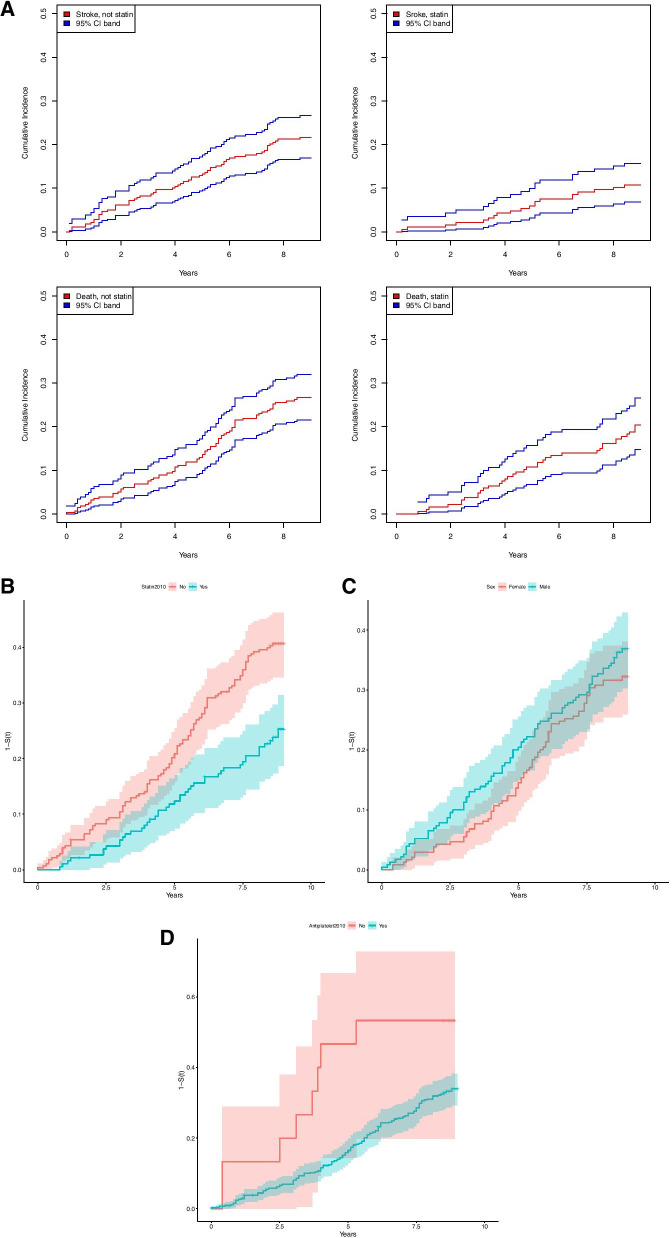
**A**. Cumulative incidence functions for recurrent cerebrovascular events. **B**. Kaplan- Meier one minus curve for all-cause mortality among patients with or without statin treatment at baseline. **C**. Kaplan- Meier one minus curve for all-cause mortality by gender. **D**. Kaplan- Meier one minus curve for all-cause mortality among patients with or without antiplatelet treatment at baseline

Advanced age was an independent predictor of recurrent stroke, CAD and all-cause mortality (Table 4). Taking death competing risk into account, the cumulative incidence function for recurrent cerebrovascular event was shown in Fig. 2A. Statin therapy at baseline had a protective effect for recurrent stroke (cs HR = 0.476, 95% CI 0.285–0.796, *P* = 0.005) and all-cause death (adjusted HR = 0.693, 95% CI 0.486–0.968, *P* = 0.043). Kaplan- Meier one minus curves for all-cause mortality among patients with or without statin treatment (Fig. 2B, *P* < 0.001), gender (Fig. 2C, *P* = 0.256), and with or without antiplatelet treatment (Fig. 2D, *P* = 0.024) at baseline were plotted. There was no statistically significant difference based on gender on Kaplan- Meier survival curve. However, Cox Proportional Hazards analysis found younger age, statin usage, female, antiplatelet treatment and a higher DBP at baseline were independent predictors for survival after adjusting for confounding factors. Age (*P* < 0.001), male (*P* = 0.016) and antiplatelet treatment (*P* = 0.004) were independent risk factors for fatal recurrent stroke. Age (*P* < 0.001), male (*P* = 0.002) and lower DBP at baseline (*P* = 0.01) were independent risk factors for fatal CAD, whereas antiplatelet treatment (*P* = 0.01) has a protective effect for fatal CAD.

**Table 4:** Cox proportional hazards model adjusting death as competing risk factor to estimate the cause- specific hazard ratio

	**Recurrent cerebrovascular event (*n* = 80)**			**CAD (*n* = 57)**			**Death (*n* = 158)**		
	**csHR**	**95% CI**	***P* value**	**csHR**	**95% CI**	***P* value**	**HR**	**95% CI**	***P* value**
**Event Type**									
**Recurrent**									
Age (year)	1.035	1.009–1.061	***0.009***	1.076	1.041–1.112	** < *0.001***	1.089	1.066–1.111	** < *0.001***
Sex (Male vs Female)	1.427	0.903–2.254	0.128	0.916	0.531–1.581	0.754	1.654	1.191–2.298	***0.003***
SBP (mmHg)	0.993	0.977–1.010	0.439	1.010	0.990–1.031	0.334	1.009	0.997–1.021	0.149
DBP (mmHg)	0.994	0.968–1.020	0.627	1.010	0.980–1.041	0.521	0.982	0.964–1.000	***0.048***
DM	1.380	0.866–2.199	0.176	0.904	0.503–1.623	0.734	1.117	0.789–1.582	0.534
Statin user	0.476	0.285–0.796	***0.005***	0.751	0.425–1.328	0.325	0.693	0.486–0.968	***0.043***
Antiplatelet treatment	1.102	0.269–4.514	0.893	1.357	0.186–9.917	0.764	0.390	0.190–0.804	***0.001***
**Death**									
Age (year)	1.102	1.074–1.130	** < *0.001***	1.080	1.056–1.105	** < *0.001***			
Sex (Male vs Female)	1.614	1.093–2.383	***0.016***	1.804	1.237–2.631	***0.002***			
SBP (mmHg)	1.004	0.989–1.012	0.599	1.003	0.990–1.018	0.601			
DBP (mmHg)	0.992	0.970–1.013	0.448	0.974	0.954–0.994	***0.010***			
DM	1.061	0.698–1.615	0.781	1.126	0.758–1.674	0.557			
Statin user	0.904	0.599–1.3626	0.628	0.733	0.491–1.095	0.129			
Antiplatelet treatment	0.314	0.144–0.687	***0.004***	0.359	0.165–0.781	***0.010***			

*CAD* indicates coronary artery disease; *csHR* cause-specific hazard ratio; *CI* confidence interval; *HR* hazard ratio; *SBP* systolic blood pressure; *DBP* diastolic blood pressure and *DM* diabetes mellitus

## Discussion

To the best of our knowledge, this is the first study to document the long-term clinical outcomes of ischemic stroke patients in primary care in Hong Kong. There were 80 patients (17.2%) whom had a recurrent stroke and 158 (33.9%) death cases during a median follow-up duration of 8.7 years. A local study [14] including 705 acute ischemic stroke patients reported that 199 (28%) suffered further cerebrovascular cardiac events and 117 patients died (17%) during a follow-up period of 42 months. A US population-based study [15] including 663 first-ever ischemic stroke patients aged 45–64 years found the 5-year recurrent rate was 14.6% and mortality rate was 19.2%, respectively. We observed a lower recurrent stroke rate because of the following reasons: 1) patients had good control of blood pressure and DM at baseline; 2) the risk of recurrent stroke was highest in the first year [3–7, 14, 15] after the index stroke, and our cohort mainly consisted of patients with chronic stroke; 3) certain subgroup of stroke patients i.e., those with cardioembolic stroke, was not well presented in our study. It is known that cardioembolic stroke survivors carries a higher risk for recurrent cardiovascular events [4, 16]. Since warfarin was not available in the drug formulary of hospital authority clinics in the 2010, only five AF patients with contraindication of anticoagulation were included in this study. The higher mortality in our study may be explained by the relatively older age at baseline as compared to both HK and US study.

At baseline, 96.8% of ischemic stroke patients were on antiplatelet treatment. However, only 40.1% of patients were receiving statin at baseline and the mean LDL level (2.9 ± 0.7 mmol/L) was significantly beyond target. Although statin plays a key role in the medical therapy of secondary stroke prevention, studies have shown lower-than-expected statin treatment rates in the primary care with a prescription rates ranging from 29–37.7% [17–19]. The inadequate use of statin in our cohort was similar to those reported in North America and Europe. This may represent a practice gap in primary care that warrant future research, as statin offers considerable reduction of CVD risk and survival benefit among these high-risk patients. The suboptimal use of statin might be due to the restricted drug formulary in public clinics in the earlier years when evidence of statin therapy was not well established. Other reason such as clinician’s knowledge of the updated guidelines of secondary prevention of stroke needs to be explored in the future studies. Some patients may overestimate their ability to comply with the required level of intensive lifestyle intervention for lipid control. It is also common that patients in our locality may rely on Chinese herbs or alternative medicines for lowering lipid level. Some patients are reluctant to start statin because of fear of the potential side effects of myalgia and liver impairment. Therefore, making a shared decision with our stroke survivors to initiate or continue stain may be challenging especially for those patients who were not receiving statin therapy before discharged from specialist clinic.

The beneficial effects of statins on reduction of cardiovascular events and improvement of survival have been well demonstrated in clinical trials [19–21]. Such evidences need to be explored under normal daily clinical practice. We observed that statin therapy reduces stroke recurrence and improves survival, which were compatible with cohorts from Spain [19] and Japan [22]. However, the baseline LDL level among patients with and without recurrent stroke was similar in our study. Our finding suggested that the beneficial effect of statin was not solely related to lowering of LDL level. The pleiotropic effects of statin included modulating inflammatory responses, ameliorating endothelial function and increasing plaque stabilization [22–24]. A local study found that use of statin leads to a significant decrease in cardiovascular events and all-cause death among Chinese diabetic patients in primary care setting [25]. The efficacy of statin could be observed even in those diabetic patients on statin treatment but did not achieve the LDL target of less than 2.6 mmol/L [25].

Our study was unable to observe the beneficial effect of statin on recurrence of ischemic heart disease, as was observed in the Stroke Prevention by Aggressive Reduction of Cholesterol Levels (SPARCL) study. This may be attributed to the small sample size in our study. Another possible explanation was the different type and dose of statin used in clinical practice. The high dose of Atorvastatin (80 mg daily) used in SPARCL study was rarely used in Asian populations. Simvastatin was the only regimen available in public clinics in 2010. The majority of our patients were using Simvastatin 10–20 mg daily. Results of Fukuoka stroke registry [22] suggested that low dose statin may reduce the risks of recurrent stroke and all-cause mortality, which was in consistency with our findings. However, the study from Japan did not report the incidence of CAD [22].

In our analysis, a higher DBP at baseline and antiplatelet treatment were independent predictors for survival. This finding was coherent with the Sweden study that antiplatelet therapy in patients with ischemic stroke had a survival benefit [26]. Lowering BP after stroke is associated with a significant decrease in stroke recurrence, but the optimal target of BP for ischemic stroke patient is uncertain [27, 28]. Meta-analysis study [29] found more intensive BP lowering further reduced stroke risk. However, it has no clear effects on cardiovascular death and total mortality [27, 28]. Results from ARIC cohort (Atherosclerosis Risk In Communities) [30] including patients without known cardiovascular disease showed that lowering of DBP to < 70 mm Hg was independently associated with more frequent CAD and death. Although the mechanism is unclear, a low DB*P* < 70 mmHg, might comprise myocardial perfusion and associate with adverse outcomes. Similarly, a low DB*P* < 70 mmHg among patients with heart failure [29] was associated with a significantly higher risk of major cardiovascular events, all-cause mortality, but not stroke. More importantly, the pooled data analysis [31] including 30,937 patients with a prior history of myocardial infarction or stroke found that a reduction of SB*P* < 120 mmHg or DB*P* < 70 mmHg was associated with an increased risk of both cardiovascular death and all-cause death, and with no risk reduction of MI or stroke. The study observed that a target SBP of 120–130 mmHg and DBP of 70–80 mmHg is associated with the lowest rates of cardiovascular disease event [32]. SBP used to be one of the main treatment targets of secondary stroke prevention protocol. As a primary care physician, we should raise awareness of the detrimental effect of excessive low DBP in our daily practice. Systolic and diastolic BP are inextricably linked, we need to consider both SBP and DBP levels while taking care of stroke survivors. While titrating antihypertensive drugs to achieve SB*P* < 140 mmHg, it may be prudent to ensure that the diastolic blood pressure does not fall below 70 mmHg [30].

Our study should be evaluated taking note of several important limitations. Firstly, this was a retrospectively study therefore unobserved potential confounders might affect the results. Secondly, despite of the 9-year follow-up, the sample size was relatively small. Our findings may not be generalizable to the entire population under primary care in HK. A territory-wide prospective cohort of ischemic stroke patients under primary care to characterize potential predictors of recurrent cerebrovascular events and mortality might be needed. Thirdly, the occurrence of cardiovascular events and death were identified by CMS record. We were not able to capture clinical outcome of patients who were admitted to private hospitals. However, the vast majority of elderly patients with chronic illness are seeking care under the public health care system in HK. It is therefore unlikely that exclusion of patients who presented to the private sector would have affected the results of the study. Fourthly, we do not have information regarding the stroke etiology and severity, so we cannot adjust for those factors although they might have an influence on long term clinical outcomes and mortality. Patients follow-up in primary care clinics usually had minor stroke with good recovery, most patients were able to walk independently or with walking aid after stroke rehabilitation**.** The etiology of index stroke was uncertain in some cases. Only about 20% of patients had underwent detailed neuroimaging studies including CT (Computed tomography) brain, MRI (Magnetic resonance imaging) brain with MRA (Magnetic resonance angiography), cervical and transcranial Doppler Ultrasonography. Therefore, we were not able to study the outcomes of different types of ischemic stroke separately. In addition, we did not have information on drug compliance of statin and the exact dose of statin taken by individual patient during the entire follow-up period. Some patients may have started statin therapy a few years after the baseline. However, the beneficial effects of statin would be underestimated under these circumstances.

## Conclusions

The long-term prognosis of ischemic stroke patients in primary care is favorable. Use of statin at baseline was associated with a significant decrease in stroke recurrence and all-cause mortality. The under-prescription of statin may represent a possible practice gap that requires future research to identify potential reasons for suboptimal use of statin in primary care. Our study also found patients who died had a significant lower DBP at baseline. It highlighted the importance of not focusing only on the level of SBP, but emphasized the need to consider both SBP and DBP levels in daily clinical practice. To optimize secondary stroke prevention in primary care, a regular evaluation of vascular risk-factor control of stroke survivals is crucial to identify and sustain improvements in taking care of our stroke patients.

## Data Availability

The datasets used and/or analyzed during the current study are available from the corresponding author on reasonable request.

## Abbreviations

HA: Hospital authority
HK: Hong Kong
TIA: Transient ischemic stroke
CAD: Coronary artery disease
csHR: Cause-Specific hazard ratio
CI: Confidence interval
HR: Hazard ratio
DBP: Diastolic blood pressure
CVD: Cardiovascular disease
NTEC: New Territories East Cluster
BMI: Body mass index
BP: Blood pressure
DM: Diabetes mellitus
LDL: Low-density lipoprotein
AF: Atrial fibrillation
GOPC: General out-patient clinic
CMS: Clinical Management System
ICPC-2: International Classification of Primary Care, second edition
ICD-9-CM: International Classification of Diseases, Ninth Edition, Clinical Modification
SBP: Systolic blood pressure
DBP: Diastolic blood pressure
SPARCL: Stroke Prevention by Aggressive Reduction of Cholesterol Levels
ARIC: Atherosclerosis Risk In Communities
CT: Computed tomography
MRI: Magnetic resonance imaging
MRA: Magnetic resonance angiography
